# A Rare Case of Bronchopulmonary Infection by Lophomonas Blattarum : A Case Report

**DOI:** 10.31729/jnma.8866

**Published:** 2025-01-31

**Authors:** Arun Kumar Mahato, Sonu Shah, Rupak K C, Sudarshan Kandel, Neetika Paudel, Saharoj Siddiqui, Niranjan K C

**Affiliations:** 1 Department of Pulmonary, Critical Care and Sleep Medicine, Nobel Medical College and Teaching Hospital, Kanchanwari, Biratnagar, Nepal; 2 Department of Internal Medicine, Nobel Medical College Teaching Hospital, Biratnagar, Morang, Nepal; 3 Maharajguni Medical Campus, Maharajgunj, Kathmandu, Nepal; 4 Department of InternarMedicine, Nepalgunj Medical College Teaching Hospital, Kohalpur, Banke, Nepal

**Keywords:** *bronchopulmonary infection*, *hepatitis B*, *immunosuppression*, *Lophomonas*, *parasite*

## Abstract

Lophomonas blattarum is a flagellated protozoan parasite found in cockroaches' and termites' hindgut. It can rarely cause bronchopulmonary infection in humans, especially in people with other comorbidities or immunocompromised, but the prevalence and characterization of the disease remains poorly understood.

In this case report, we present a case of a 51-year-old male patient with underlying Hepatitis B presenting with a persistent cough for more than two weeks. During evaluation, microscopic examination of the wet mount of Bronchoalveolar lavage revealed actively motile trophozoite of Lophomonas blattarum. The patient was treated with metronidazole with significant improvement in two weeks.

## INTRODUCTION

Lophomonas blattarum is a flagellated protozoan parasite which is found in the intestinal tracts of cockroaches.^1^ Some arthropods including termites also harbor Lophomonas in their hindgut. It lives in symbiosis with cockroaches and is rarely reported as a pathogen in humans. It has only trophozoite form in humans while it can demonstrate both trophozoite and cyst stages in the insect hosts.^1^ Though disease due to Lophomonas is rare, it primarily causes bronchopulmonary infections and sometimes upper respiratory tract disease. Airborne transmission of Lophomonas occurs due to inhalation of aerosolized cysts. The symptoms commonly mimic pneumonia. It consists of productive or non-productive cough, chest pain, shortness of breath, signs of respiratory failure, etc. It usually occurs in patients with compromised immune status.^2^ Patient can also be asymptomatic or may have a chronic infection or co-infection with Tubercular bacilli, HIV, hydatid cyst, etc.^3^

## CASE REPORT

A 51-year-old male presented to our center on 2nd July 2024 with complaints of a cough that was initially dry for 20 days then the cough was blood-mixed, along with throat irritation for 7 days. There was a history of weight loss. There was no previous history of such episodes or any other significant illness. He had been suffering this for 1 month for which he visited the nearby provincial hospital and then presented to Nobel Medical College and Teaching Hospital.

**Figure 1:**
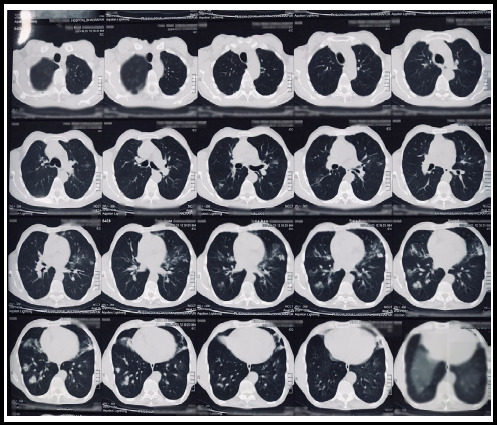
Non-Contrast Computed Tomography (CT) of the chest showing few discrete ground glass nodules in bilateral lungs predominantly right lower and middle lobes and left upper lobe.

In the provincial hospital, routine laboratory investigations revealed Hemoglobin-10 gm/dl with normal white blood cell and eosinophil count. The erythrocyte sedimentation rate was 10 mm fall in the first hour. A sputum smear for acid-fast bacilli (AFB) was sent and found to be negative. CT scan of the chest revealed a few discrete ground glass nodules in bilateral lung fields predominantly in the right lower and middle lobes and left upper lobe suggestive of infective pathology.(Figure 1) Upon this finding of the CT chest, a differential diagnosis of pneumonia was made and oral antibiotics with conservative management were done. But due to no resolving of symptoms mainly blood in sputum, he then visited our center for further evaluation and management.

**Figure 2:**
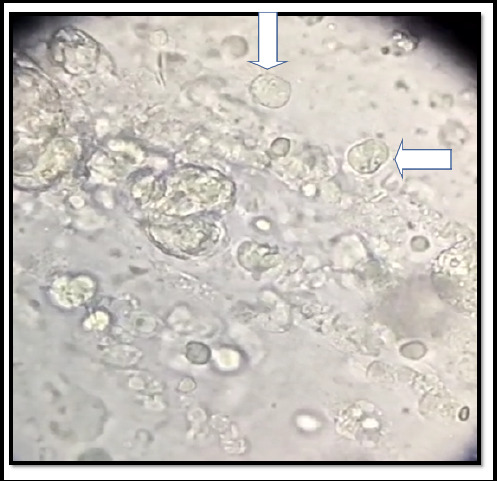
Microscopic examination of the wet mount of Bronchoalveolar lavage showing actively motile flagellates with a polar tuft of flagella lashing rhythmically, identified as trophozoite of Lophomonas blattarum as shown by the arrow.

On 2nd July 2024, he presented to the Pulmonology outpatient department after 7 days of his initial medication with the above-mentioned symptoms. Upon taking history he gives a history of living in old houses for many years where cockroach infestation was common, especially in kitchen areas. On examination, his vital signs were: Pulse rate 62 beats/min, blood pressure 120/72 mmHg, respiratory rate 20 breaths/min, and body temperature 36.5°C. Systemic examination showed normal vesicular breath sound on bilateral lung field auscultation. The clinical laboratory tests for blood, urine, feces, and hepatic and renal functions were within normal limits. On considering the age factor, weight loss, and symptoms a repeat sputum smear for acid-fast bacilli (AFB) was sent along with Gene Xpert and Serology with a suspect of pulmonary tuberculosis. Again the sputum smear for AFB was negative along with Gene Xpert. Serology revealed hepatitis B virus surface antigen (HBsAg) to be positive whereas human immunodeficiency virus (HIV), and hepatitis C antibody (anti-HCV) tests were negative. So now Fiberoptic bronchoscopy and bronchoalveolar lavage (BAL) were done to evaluate discrete lung nodules with respiratory symptoms. Bronchoscopy also showed normal study. Finally, BAL was taken and then sent for microscopic examination and cytology. Microscopic examination of the wet mount of BAL revealed actively motile flagellates with a polar tuft of flagella lashing rhythmically, identified as trophozoite of L. Blattarum. (Figure 2)

The patient was then admitted to the pulmonology ward. Oral metronidazole 400 mg thrice daily was started along with other conservative medications. Steady improvement in the patient's condition was noted, and so was discharged after 2 days of admission with continuation of oral metronidazole for 2 weeks. On follow-up his symptoms were all resolved, becoming asymptomatic after 2 weeks of treatment. A Repeat Chest x-ray was done which showed normal findings.

## DISCUSSION

There is an increasing number of case reports of infection by Lophomonas blattarum with associated clinical and radiological findings over the past decade. As per a report published in 2022, Majority number of cases (94.4%) have been documented among Chinese population.^3^ A recent systematic review of studies done excluding articles in Chinese language documented 307 cases of Lophomonas infection between 1993 and 2020 and the most reported instances were from Iran followed by China, Panama, Turkey, India, etc. Highest number of cases (55.7%) were reported in the age group less than 18 years old.^4^ To our knowledge, no cases of Lophomonas has been reported from Nepal till date. Though cases reported are only from a few countries, it may not be restricted to specific geographical locations and can be found in the places where they have not previously been documented. Lack of awareness about the likelihood of protozoal lung infection in certain patient population and characteristics signs as well as lack of routine microscopic observation of fresh sputum leads to under recognition of lophomonas.^2^

The clinical manifestations and radiological findings of our patient were similar to those mentioned in other published literatures. Most of the patients of bronchopulmonary infection by Lophomonas have prior history of exposure to termite and cockroaches or they live in old houses in urban places. In our case too, the patient has been living in old houses for many years where cockroach infestation is common especially in kitchen areas. Likely, he was exposed to the pathogen at his own home.

The clues to the diagnosis of this infection are respiratory symptoms in immunocompromised subjects or those patient on immunosuppressant drug for a long period of time, eosinophilia, poor antibiotic response. The persistent cough, poor antibiotic response with negative sputum examination and culture in this patient with active Hepatitis B as a comorbidity encouraged the further evaluation of patient by bronchoscopy and microscopic examination of bronchoalveolar lavage helped to diagnose the causative agent Lophomonas Blattarum. One case was identified in immunocompetent young individual in India similar to our case except the age factor and comorbidity. Nitro-imidazole antimicrobials like metronidazole has been used as antiprotozoal therapy for bronchopulmonary Lophomoniasis and has been found to produced satisfactory outcomes.^5^ Our patient was also treated with Metronidazole after diagnosis and significant improvement in his condition was observed in two weeks.

In conclusion, bronchopulmonary infection caused by Lophomonas in a Hepatitis B patient represents a rare, and yet clinically significant occurrence. This highlights the importance of considering parasitic infection in individuals with respiratory symptoms who have compromised immune status, especially in patients living in places with cockroach infestation. This shows the need for further research to better understand the etiology, pathogenesis and optimal treatment strategies.
